# TonEBP Regulates PCNA Polyubiquitination in Response to DNA Damage through Interaction with SHPRH and USP1

**DOI:** 10.1016/j.isci.2019.07.021

**Published:** 2019-07-19

**Authors:** Hyun Je Kang, Hyun Park, Eun Jin Yoo, Jun Ho Lee, Soo Youn Choi, Whaseon Lee-Kwon, Kyoo-young Lee, Jin-Hoe Hur, Jeong Kon Seo, Jae Sun Ra, Eun-A. Lee, Kyungjae Myung, Hyug Moo Kwon

**Affiliations:** 1 School of Life Sciences, Ulsan National Institute of Science and Technology, Ulsan 44919, Republic of Korea; 2 Center for Genomic Integrity, Institute for Basic Science, Ulsan 44919, Republic of Korea; 3 UNIST-Optical Biomed Imaging Center (UOBC), Ulsan National Institute of Science and Technology, Ulsan 44919, Republic of Korea; 4 UNIST Central Research Facilities (UCRF), Ulsan National Institute of Science and Technology, Ulsan 44919, Republic of Korea

**Keywords:** Biological Sciences, Biochemistry, Molecular Biology, Cell Biology

## Abstract

Polyubiquitination of proliferating cell nuclear antigen (PCNA) regulates the error-free template-switching mechanism for the bypass of DNA lesions during DNA replication. PCNA polyubiquitination is critical for the maintenance of genomic integrity; however, the underlying mechanism is poorly understood. Here, we demonstrate that tonicity-responsive enhancer-binding protein (TonEBP) regulates PCNA polyubiquitination in response to DNA damage. TonEBP was recruited to DNA damage sites with bulky adducts and sequentially recruited E3 ubiquitin ligase SHPRH, followed by deubiquitinase USP1, to DNA damage sites, in correlation with the dynamics of PCNA polyubiquitination. Similarly, TonEBP was found to be required for replication fork protection in response to DNA damage. The Rel-homology domain of TonEBP, which encircles DNA, was essential for the interaction with SHPRH and USP1, PCNA polyubiquitination, and cell survival after DNA damage. The present findings suggest that TonEBP is an upstream regulator of PCNA polyubiquitination and of the DNA damage bypass pathway.

## Highlights

• TonEBP regulates MMS-induced PCNA polyubiquitination
• TonEBP interacts with SHPRH and USP1
• TonEBP recruits SHPRH and USP1 to DNA damage sites
• TonEBP reduces DNA damage-induced mutagenesis and cell death

## Graphical Abstract

**Figure:**
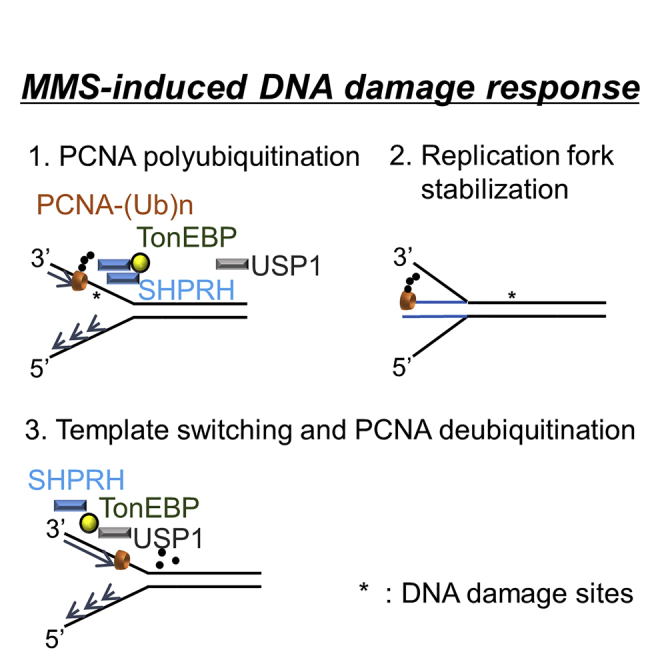

## Introduction

DNA damage, such as bulky adducts, causes DNA polymerase stalling. DNA damage tolerance mechanisms allow for a bypass of DNA lesions to suppress the collapse of the DNA replication fork (Berti and Vindigni, 2016). Two DNA damage tolerance mechanisms have been identified: translesion synthesis and template switching. In translesion synthesis, error-prone translesion DNA polymerases replace replicative DNA polymerases in response to proliferating cell nuclear antigen (PCNA) monoubiquitination at the expense of the increased mutation rate (Berti and Vindigni, 2016, Kannouche and Lehmann, 2004). Template switching is initiated by polyubiquitination of PCNA and is an error-free pathway of DNA lesion bypass mediated by an uncharacterized mechanism.

PCNA polyubiquitination in mammals is catalyzed by ubiquitin E3 ligases SHPRH and HLTF (Motegi et al., 2006, Motegi et al., 2008, Unk et al., 2006, Unk et al., 2008), whereas PCNA deubiquitination is catalyzed by USP1 (Huang et al., 2006). There are two potential models explaining the role of PCNA polyubiquitination in the DNA damage bypass. In one model, PCNA polyubiquitination facilitates filling of postreplicative single-stranded DNA (ssDNA) gaps via template switching and recombinational mechanisms involving sister chromatid junctions (Berti and Vindigni, 2016, Giannattasio et al., 2014). An alternative model of error-free bypass and template switching entails remodeling of the replication fork in a four-way junction, a process known as replication fork reversal, to enable template switching to occur directly in the elongating fork (Higgins et al., 1976).

SHPRH is a mammalian homolog of *S. cerevisiae* Rad5. SHPRH polyubiquitinates PCNA to promote DNA lesion bypass via an unknown recombination-dependent pathway (Motegi et al., 2006, Unk et al., 2006). These functions of SHPRH are mediated by interactions with PCNA, RAD18, and UBC13. USP1 regulates several important steps in DNA damage response, mainly in the Fanconi anemia (FA) pathway and in the process of translesion synthesis (Huang et al., 2006, Nijman et al., 2005). FA is a genomic instability disorder caused by mutations in genes regulating the replication-dependent removal of interstrand DNA cross-links (D'Andrea and Grompe, 2003, Kais et al., 2016). Recent evidence suggests that USP1 contributes to the regulation of differentiation in specific cellular contexts. USP1 is activated by forming a heterodimeric complex with its cofactor USP1-associated factor 1 (UAF1) (Cohn et al., 2009).

Tonicity-responsive enhancer-binding protein (TonEBP), also known as nuclear factor of activated T cells 5 (NFAT5), belongs to the Rel family of DNA-binding transcription factors, which includes nuclear factor (NF)-κB and NFAT (Miyakawa et al., 1999). TonEBP is involved in a variety of processes including transcriptional regulation and transcriptional stimulation through sequence-specific DNA binding (Miyakawa et al., 1999) and acts as a transcriptional cofactor of NF-κB (Lee et al., 2016a) and a transcriptional suppressor of genes *PPARγ* and *IL-10* (Choi et al., 2016, Lee et al., 2015). TonEBP upregulation is crucial in inflammatory diseases, including rheumatoid arthritis (Yoon et al., 2011), atherosclerosis (Halterman et al., 2012), and diabetic nephropathy (Choi et al., 2018). TonEBP upregulation promotes hepatocellular carcinogenesis (Lee et al., 2019). According to our present data, the function of TonEBP in DNA damage response is unrelated to the regulation of transcription.

Protein interactome studies help to identify new functions of proteins of interest (Feng et al., 2016, Gingras et al., 2007, Lee et al., 2016b, Scott et al., 2017). TonEBP is similar to PCNA in terms of the mechanism of DNA encirclement. Because PCNA is an important factor in DNA metabolism, proteomic analysis was performed here to determine whether TonEBP is also involved in DNA metabolism. In these experiments, we identified interactions of TonEBP with SHPRH and USP1.

In the present study, we showed that TonEBP functions as an early sensor of the DNA damage response. TonEBP was found to be recruited to DNA damage sites with bulky adducts and to regulate PCNA polyubiquitination through sequential interactions with SHPRH and USP1. Accordingly, TonEBP suppressed cell death caused by mutagenesis in response to DNA damage.

## Results

### TonEBP Is Recruited to DNA Damage Sites and Interacts with Proteins Involved in PCNA Polyubiquitination

**Figure 1:**
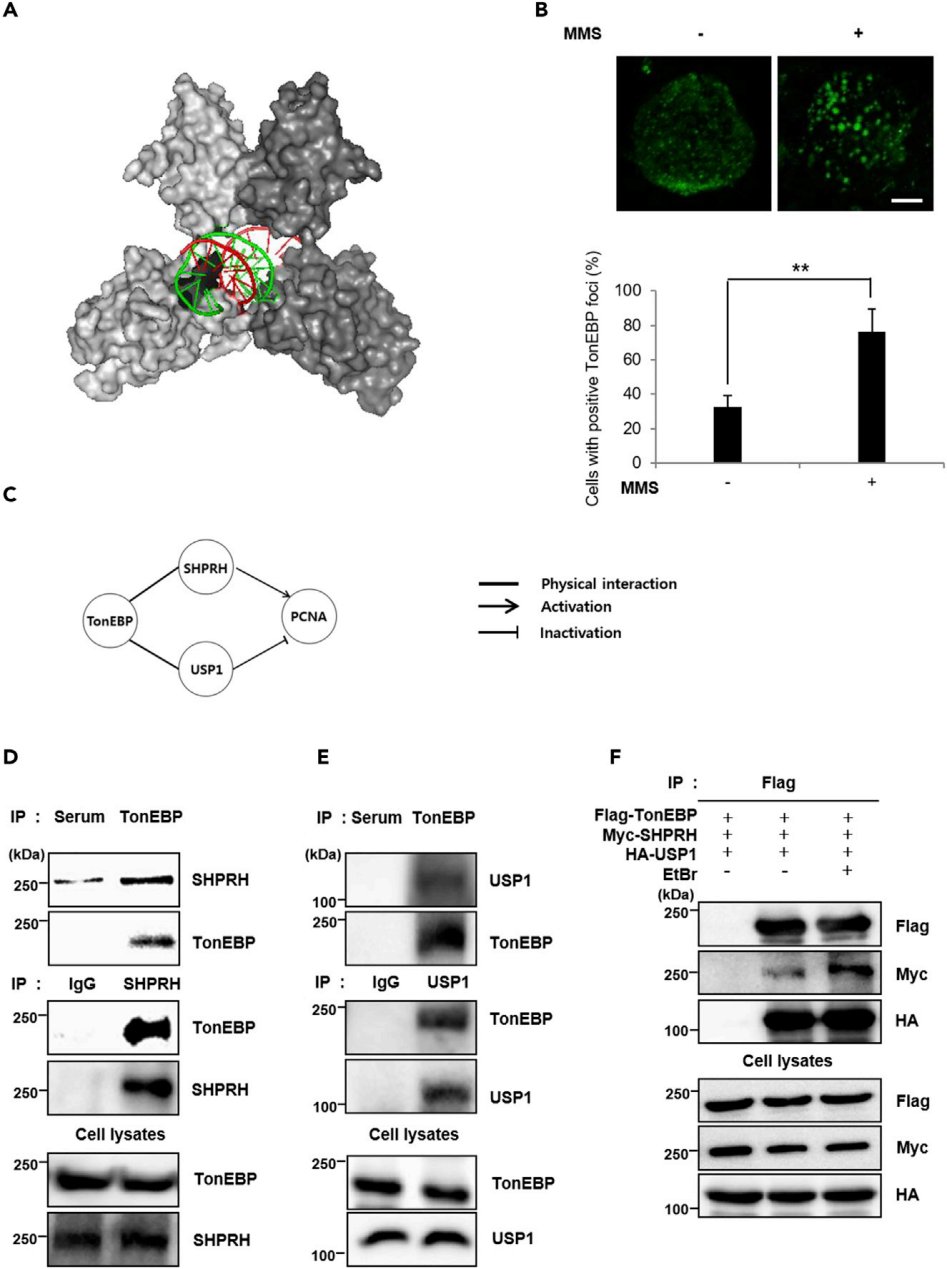
TonEBP Forms Foci in Response to DNA Damage and Interacts with SHPRH and USP1 (A) Crystal structure of a homodimer of the Rel-homology domain (RHD) of TonEBP. The DNA-binding pocket is shown in the center (PDB: 1IMH). (B) HeLa cells treated with methyl methanesulfonate (MMS, 0.01%) for 30 min were fixed and immunostained for TonEBP. Top: Representative image of a nucleus in each condition. Bottom: The percentage of cells with TonEBP foci (out of 150 nuclei) expressed as the mean ± SD, n = 3; **p < 0.01. Scale bar, 2 μm. (C) The TonEBP interactome of the PCNA ubiquitination pathway including SHPRH and USP1 (according to String v.9.1). (D and E) HEK293 cell lysates were immunoprecipitated (IP) with the indicated antibodies. Cells treated with normal serum (Serum) served as the negative control. An anti-TonEBP antibody (TonEBP) (top) and anti-SHPRH (SHPRH) antibody (middle) were used for IP (D). An anti-TonEBP antibody (top) and anti-USP1 antibody (USP1) (middle) were used for IP (E). Precipitated proteins were detected with the indicated antibodies. (F) HEK293 cells were transfected with plasmids expressing proteins FLAG-TonEBP, Myc-SHPRH, and HA-USP1. Cell lysates were immunoprecipitated with an anti-FLAG antibody. Precipitated proteins were detected with the indicated antibodies. EtBr (at 0.1 mg/mL) was added during the IP reaction to eliminate indirect interactions through DNA.

TonEBP binds to its cognate DNA sequence using the Rel-homology domain (RHD) similar to the RHD of transcription factors NFAT and NF-κB. The RHD of TonEBP forms a homodimer that completely encircles DNA, thereby creating a protein ring (Figure 1A) (Stroud et al., 2002). Because the inner diameter of the protein ring is larger than the outer diameter of DNA, the DNA surface is not in full contact with the protein in the circle. These unusual features of the TonEBP RHD point to a possible function of TonEBP in DNA surveillance. To test this possibility, the subcellular localization of TonEBP was monitored after treatment with a DNA-damaging agent. Exposure to the alkylating agent methyl methanesulfonate (MMS) for 30 min resulted in the localization of TonEBP at the sites of DNA damage and the formation of damage-induced foci (Figure 1B), suggesting that TonEBP helps to detect DNA damage. TonEBP foci formation was induced only by MMS, and not by UV light, HU (hydroxyurea), or mitomycin C (Figure S1A).

To identify the DNA repair pathway involving TonEBP, TonEBP-interacting proteins were found by screening via tandem affinity purification. We identified 465 proteins that interacted with the N terminus of TonEBP (Yc1), which encompasses the entire RHD, including known DNA repair proteins SHPRH (ubiquitin E3 ligase) and USP1 (deubiquitinase) (Figure 1C and Table S1). SHPRH (Motegi et al., 2006, Unk et al., 2006) and USP1 (Huang et al., 2006) regulate PCNA ubiquitination in response to DNA damage during DNA replication in the template-switching pathway.

### TonEBP Interacts with SHPRH and USP1 via Its RHD

To confirm the interaction between TonEBP and SHPRH or USP1, endogenous TonEBP and SHPRH were coimmunoprecipitated (Figure 1D). Immunoprecipitation (IP) with an anti-TonEBP or anti-SHPRH antibody coprecipitated SHPRH or TonEBP, respectively. This interaction was confirmed using ectopically expressed TonEBP and SHPRH (Figure S1B). The interactions of USP1 and UAF1, an essential cofactor of USP1, with TonEBP were confirmed by coimmpunoprecipitation (coIP) (Figures 1E, S1C, and S1E). Despite the opposite effects of SHPRH and USP1 on PCNA ubiquitination, they interacted with each other (Figure S1D). To rule out an indirect interaction between TonEBP and SHPRH or USP1 through DNA, whole-cell extracts were preincubated with ethidium bromide (EtBr, which intercalates DNA) or benzonase, which degrades DNA before coIP. Preincubation with EtBr or benzonase did not affect the interaction between TonEBP and SHPRH or USP1 (Figures 1F and S1F). Collectively, these results indicated that TonEBP interacts with ubiquitin E3 ligase SHPRH and deubiquitinating enzyme complex USP1–UAF1, suggesting that TonEBP participates in PCNA polyubiquitination.

**Figure 2:**
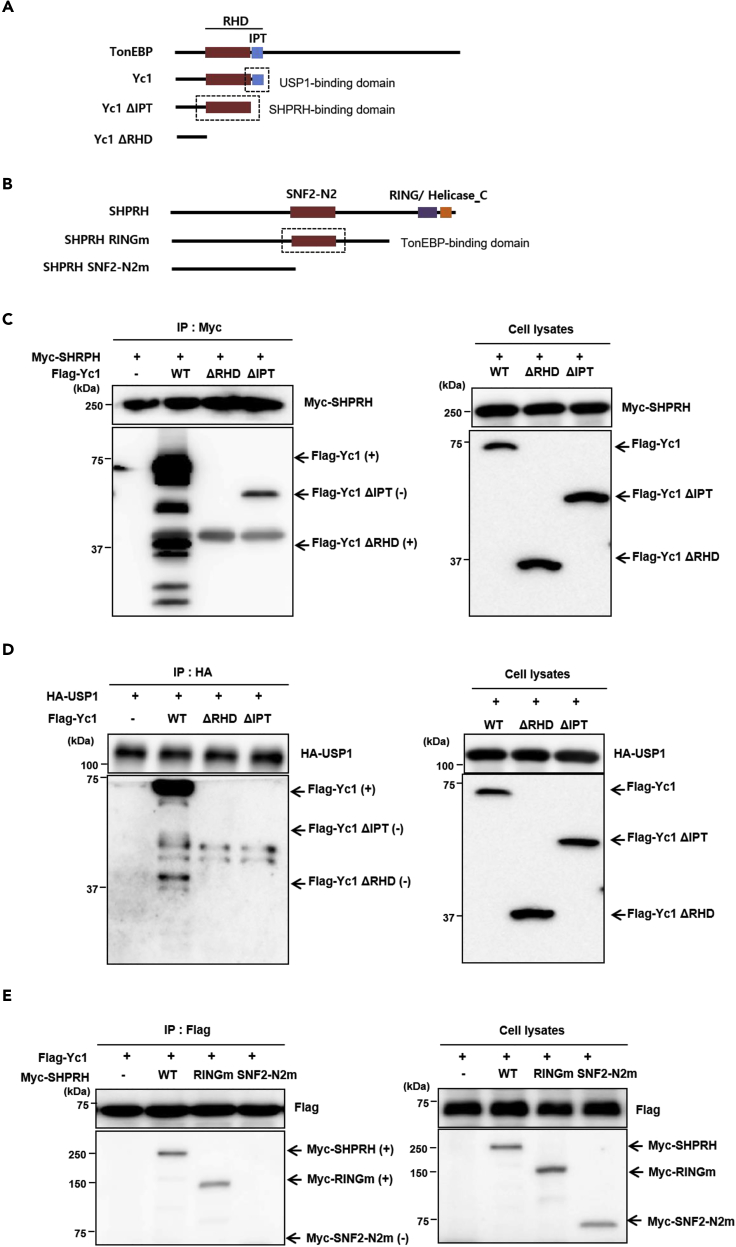
The RHD of TonEBP Is Required for the Interaction with SHPRH and USP1 (A) A schematic of the domain structures of full-length (FL) human TonEBP and the serial deletion constructs Yc1, Yc1 ΔIPT, and Yc1 ΔRHD. (B) A schematic of the domain structures of FL human SHPRH and the serial deletion constructs SHPRH RINGm and SHPRH SNF2-N2m. (C) HEK293 cells were transfected with plasmids expressing Myc-tagged SHPRH together with FLAG-tagged Yc1, Yc1 ΔIPT, or Yc1 ΔRHD. Proteins immunoprecipitated with an anti-Myc antibody were detected with the anti-FLAG antibody. (D) HEK293 cells were transfected with a plasmid expressing HA-USP1 together with a plasmid expressing FLAG-tagged Yc1, Yc1 ΔIPT, or Yc1 ΔRHD. Proteins immunoprecipitated with an anti-hemagglutinin (HA) antibody were detected with the anti-FLAG antibody. (E) HEK293 cells were transfected with a plasmid expressing FLAG-Yc1 together with a plasmid expressing Myc-tagged FL SHPRH, RINGm SHPRH, or SNF2-N2m SHPRH. Proteins immunoprecipitated with the anti-FLAG antibody were detected with the anti-Myc antibody.

To determine the domains involved in the interaction between TonEBP and SHPRH, serial deletion mutants of proteins TonEBP and SHPRH (Figures 2A and 2B) were generated and their interactions were examined. CoIP with the serial deletion mutants of TonEBP revealed that the RHD of TonEBP was required for the interaction with SHPRH, whereas the C-terminal portion of RHD named IPT was dispensable (Figure 2C). The interaction of TonEBP with USP1 required the entire RHD including IPT (Figure 2D), suggesting that SHPRH and USP1 interact with TonEBP through different sections of the RHD. The SNF2-N2 domain of SHPRH was required for the interaction with TonEBP (Figure 2E). These data indicated that TonEBP interacts with SHPRH and USP1 through distinct sections of the RHD. It is possible that the Yc1 fragment of TonEBP dimerizes with endogenous full-length TonEBP. To rule out this possibility, we depleted TonEBP expression before interaction analysis. TonEBP depletion did not affect the interaction among Yc1, SHPRH, and USP1 (Figures S2A and S2B).

### TonEBP Regulates MMS-Induced PCNA Polyubiquitination through Recruitment of SHPRH and USP1 to DNA Damage Sites via Dynamic Interactions

**Figure 3:**
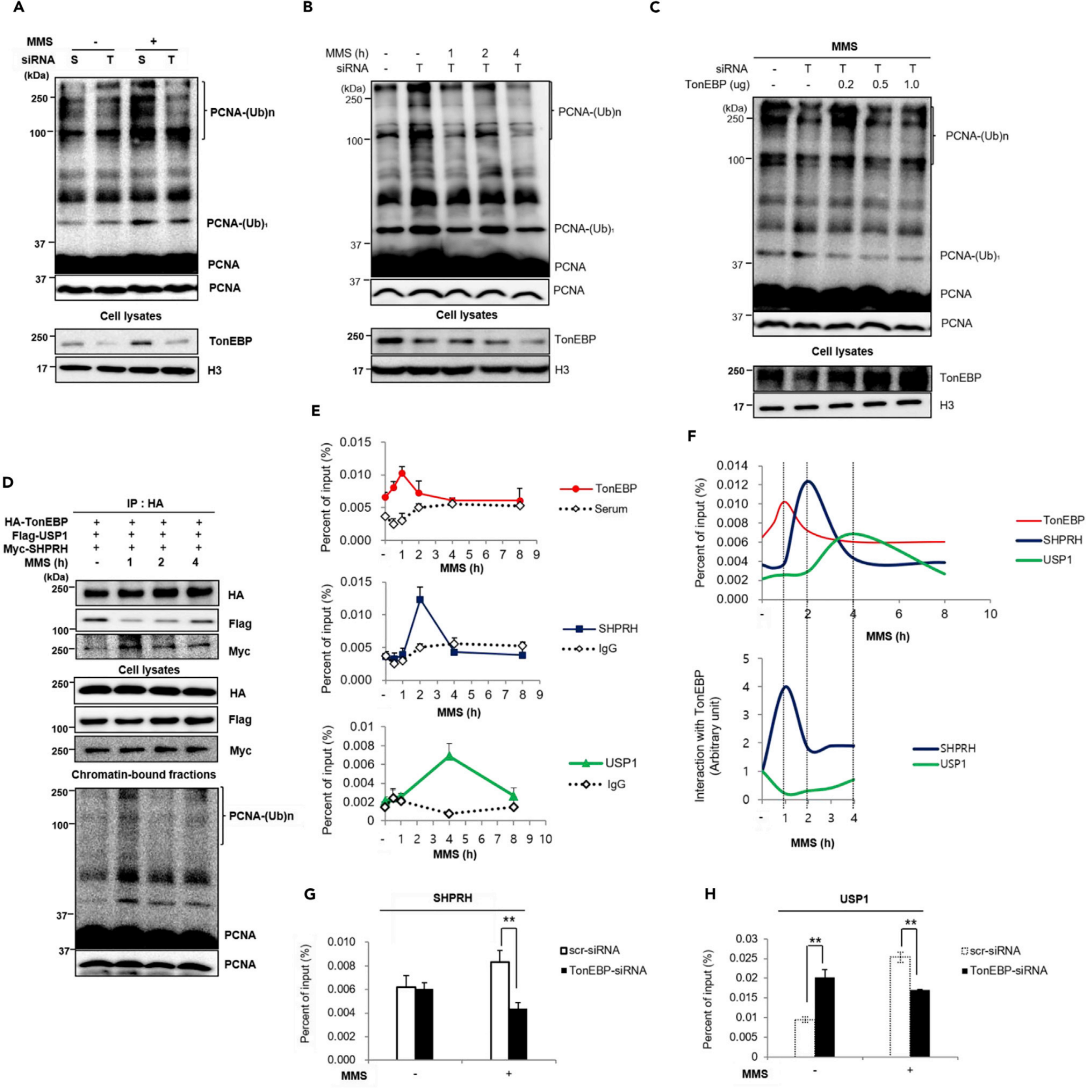
Dynamic Interactions between TonEBP and SHPRH or USP1 on DNA Damage Sites Regulate PCNA Polyubiquitination (A) HEK293 cells transfected with control (scrambled) siRNA (S) or TonEBP-targeting siRNA (T) were treated with 0.01% (v/v) MMS for 1 h as indicated. PCNA bound to chromatin was immunoprecipitated and detected with an anti-PCNA antibody (top) after removal of MMS in 4 h. PCNA-(Ub)_n_ indicates polyubiquitinated PCNA. Cell lysates were also immunoblotted (bottom). (B) HEK293 cells transfected with TonEBP-targeting siRNA (T) were treated with 0.01% (v/v) MMS for 1 h. PCNA bound to chromatin was immunoprecipitated and detected with an anti-PCNA antibody after removal of MMS according to the indicated timeline. (C) HEK293 cells transfected with TonEBP-targeting siRNA (T) and with plasmids expressing TonEBP at various doses (as indicated) were treated with 0.01% (v/v) MMS for 1 h. PCNA bound to chromatin was immunoprecipitated and detected with the anti-PCNA antibody. (D) HEK293 cells transfected with plasmids expressing hemagglutinin (HA)-TonEBP, FLAG-USP1, and Myc-SHPRH were treated with 0.01% (v/v) MMS for 1 h. Cells without MMS treatment served as the negative control (−). Cells treated with MMS were washed with a fresh medium and incubated for 1–4 h as indicated. Cell lysates from each time point were immunoprecipitated with the anti-HA antibody, and the indicated proteins were detected with each antibody. Chromatin-bound fractions were prepared separately, and PCNA was detected in the same way as in Figure 3A (bottom). (E) Cells treated with 0.01% (v/v) MMS were washed and incubated for up to 9 h as indicated. ChIP was performed using the anti-TonEBP, anti-SHPRH, or anti-USP1 antibody as indicated. Region 13502–13724 (nucleotide positions) in intron 7 of *TP53* from precipitated DNA was amplified by quantitative PCR. The percentage of input in the precipitate is shown as the mean ± SD, n = 3. (F) Top: The results in (E) were fitted to curves. Bottom: Interactions of TonEBP with SHPRH and USP1 shown in (D) were formatted as a curve. (G and H) HEK293 cells transfected with either scrambled (control) or TonEBP siRNA were treated with 0.01% (v/v) MMS. DNA precipitated by ChIP with the anti-SHPRH (G) or anti-USP1 (H) antibody was amplified by quantitative PCR after 2 or 4 h, respectively, with the same primers as those used in Figure 3F. Mean ± SD, n = 4; **p < 0.01.

SHPRH ubiquitinates PCNA, whereas USP1 deubiquitinates PCNA, for the template-switching DNA damage bypass pathway (Figures S3A and S3B) (Motegi et al., 2008, Vujanovic et al., 2017). We hypothesized that TonEBP regulates PCNA ubiquitination in response to DNA damage by interacting with SHPRH and USP1. To test this hypothesis, the effect of a TonEBP knockdown on MMS-induced PCNA polyubiquitination was assessed. PCNA polyubiquitination was increased by the TonEBP knockdown (Figures 3A and 3B). Nonetheless, the TonEBP knockdown reduced PCNA polyubiquitination in response to MMS treatment (Figure 3A). When we monitored PCNA polyubiquitination after removing MMS, PCNA polyubiquitination decreased in a time-dependent manner in the TonEBP knockdown group (Figure 3B), thereby explaining the attenuation of MMS-induced PCNA polyubiquitination in the TonEBP knockdown group compared with scrambled control groups as shown in Figure 3A. On the basis of these observations, we hypothesized that TonEBP recruits SHPRH and USP1 to damaged DNA in a dose-dependent manner. To test this hypothesis, TonEBP was expressed to various degrees, and PCNA polyubiquitination was monitored. At low TonEBP expression after transfection of 0.2 μg of the expression plasmid, PCNA polyubiquitination was induced. In contrast, transfection of 1 μg of the TonEBP expression plasmid reduced PCNA polyubiquitination (Figure 3C). SHPRH or USP1 depletion at low or high TonEBP expression, respectively, abrogated the changes of PCNA polyubiquitination by TonEBP (Figures S4A and S4B). SHPRH or USP1 were found to cause the changes in PCNA polyubiquitination at low or high TonEBP expression, respectively. To rule out the possibility that changes of PCNA polyubiquitination by TonEBP expression are due to the changes of SHPRH or USP1 expression by the TonEBP knockdown, we measured the expression of SHPRH and USP1 upon TonEBP knockdown. This knockdown did not affect the levels of SHPRH and USP1 either in the no-damage or in the MMS treatment group (Figures S5A and S5B). We additionally checked TonEBP function in UV light-induced PCNA polyubiquitination. Although PCNA polyubiquitination was mildly induced by UV light, TonEBP knockdown reduced UV light-induced PCNA polyubiquitination (Figure S6A). HLTF, an E3 ubiquitin ligase involved in UV light-induced PCNA ubiquitination (Motegi et al., 2008), interacted with TonEBP (Figure S6B). Given that SHPRH and HLTF interact with each other (Lin et al., 2011), we tested whether the interaction between TonEBP and HLTF is mediated by SHPRH. SHPRH knockdown reduced the interaction between TonEBP and HLTF (Figure S6C), suggesting that UV-induced PCNA polyubiquitination is regulated by TonEBP-SHPRH-HLTF interaction.

The regulation of PCNA polyubiquitination by TonEBP could be mediated by the activation of SHPRH or suppression of USP1 because TonEBP interacts with SHPRH and USP1 (Figures 1D and 1E). High ectopic expression of TonEBP suppressed PCNA ubiquitination in response to MMS treatment (Figure 3C). Both TonEBP knockdown and TonEBP overexpression decreased PCNA polyubiquitination, suggesting that TonEBP modulates PCNA ubiquitination by recruiting SHPRH or USP1 to DNA damage sites. To test this hypothesis, we investigated the temporal interactions of TonEBP with SHPRH and USP1 after DNA damage. Coexpression of TonEBP, SHPRH, and USP1 resulted in the reciprocal coIP of USP1 or SHPRH (Figure S7), suggesting that the three proteins form a complex. Nevertheless, assessment of the temporal pattern of these interactions in response to MMS treatment and in comparison with the timeline of PCNA ubiquitination showed an increased interaction between TonEBP and SHPRH at 1 h after MMS treatment, concomitantly with a decreased interaction between TonEBP and USP1 (Figure 3D). The interaction between TonEBP and SHPRH started to decrease at 2 h after the removal of MMS, whereas the interaction between TonEBP and USP1 increased at 4 h after the removal of MMS. Consistent with these findings, PCNA ubiquitination peaked at 1 h and declined after MMS treatment. These results indicated that TonEBP regulates PCNA polyubiquitination through dynamic temporal interactions with SHPRH and USP1 in response to MMS treatment.

To independently confirm the role of TonEBP in the recruitment of SHPRH and USP1 to DNA damage sites through dynamic interaction changes, protein recruitment to defined sites of DNA damage was measured by chromatin immunoprecipitation (ChIP). Nucleotides 13502–13724 in intron 7 of *TP53* contain well-characterized DNA damage sites (Figure S8A) (Jiang and Sancar, 2006). To prove that DNA was damaged in our DNA-damaging condition, localization of RPA32 and ATR to the *TP53* target region was next monitored by ChIP analysis. RPA32 and ATR were recruited to the *TP53* target region at 30 min and 1 h after MMS treatment, respectively (Figure S8B). TonEBP recruitment to this region peaked at 1 h after MMS treatment (Figures 3E and S8C). By contrast, the recruitment of SHPRH and USP1 to the region peaked at 2 and 4 h after MMS treatment, respectively (Figure 3E and the top of Figure 3F). This sequential pattern of recruitment to the DNA damage site was consistent with the pattern of interactions of SHPRH and USP1 with TonEBP (Figure 3E and the bottom of Figure 3F). SHPRH and USP1 recruitment decreased in response to small interfering RNA (siRNA)-mediated silencing of TonEBP expression (Figures 3G and 3H), suggesting that TonEBP recruited both SHPRH and USP1 to DNA damage sites.

### TonEBP Protects the Replication Fork in Response to MMS Treatment

**Figure 4:**
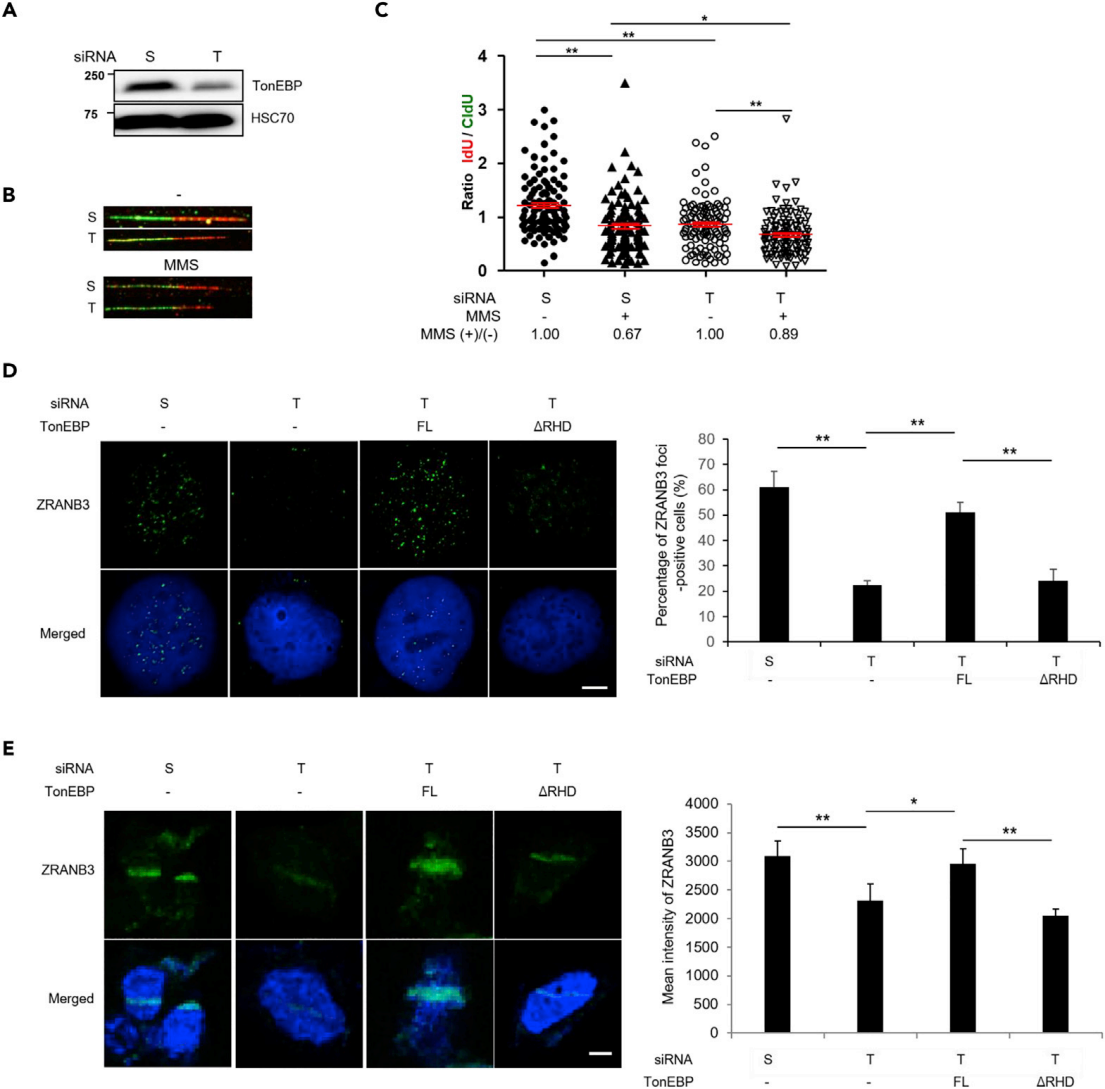
TonEBP Promotes Replication Fork Reversal (A) The TonEBP protein level was determined by western blot analysis. HEK293 cells transfected with scrambled siRNA (S) or TonEBP-targeting siRNA (T). Whole-cell extracts were subjected to western blot analysis. (B) Representative images of DNA fibers are shown. (C) Analysis of the replication fork reversal in a DNA combing assay. The iodo-deoxyuridine/chloro-deoxyuridine (IdU/CIdU) ratio was measured in at least 200 well-isolated DNA fibers from three independent experiments; mean ± SD, *p < 0.05, **p < 0.01. (D and E) U2OS cells transfected with TonEBP-targeting siRNA (T) together with a plasmid expressing tagged Yc1, Yc1 ΔIPT, or Yc1 ΔRHD. U2OS cells irradiated with UV light (60 J/m^2^) (D) or microirradiated with 405-nm UV light (E) were fixed and immunostained with the anti-ZRANB3 antibody. Right: The percentage of cells with ZRANB3 foci (D) and intensity of UV light-induced ZRANB3 stripes (out of 50 nuclei) (E) expressed as the mean ± SD, n = 3; *p < 0.05, **p < 0.01. Scale bar, 2 μm.

Because PCNA polyubiquitination promotes replication fork reversal in response to DNA damage (Vujanovic et al., 2017), we next hypothesized that TonEBP regulates replication fork reversal. A DNA combing assay can detect fork reversal impairment by measuring unrestrained fork progression (Vujanovic et al., 2017). The replication velocity depended on TonEBP without replication stress. Nonetheless, TonEBP depletion caused a smaller reduction of replication velocity in response to MMS treatment (Figures 4A–4C). We additionally confirmed the specificity of an anti-ZRANB3 antibody by means of siRNA targeting ZRANB3 (Figure S9A). Native 5-bromo-2′-deoxyuridine (BrdU) detection (without DNA denaturation) immediately after BrdU labeling for 5 min detects ssDNA upon DNA damage, including reversed forks (Couch et al., 2013). The intensity of BrdU foci increased in response to MMS treatment, consistent with another report (Nikolova et al., 2010) (Figures S9B and S9C). In agreement with the PCNA polyubiquitination attenuation by the TonEBP knockdown, the intensity of native BrdU foci decreased. Following DNA damage, a smaller reduction in fork velocity and slower single-stranded DNA accumulation after the TonEBP knockdown suggested that TonEBP might promote the reversal of DNA replication forks. The ZRANB3 DNA translocase is required for replication fork reversal upon DNA damage (Ciccia et al., 2012). In line with the above data, the TonEBP knockdown decreased UV-induced ZRANB3 foci and ZRANB3 recruitment to DNA damage sites (Figures 4D and 4E). In addition, we examined the importance of the TonEBP RHD, which encircles DNA and is essential for the interaction with SHPRH or USP1 (Figure 1A). Full-length (FL) and Yc1 TonEBP restored UV-induced ZRANB3 focus formation and ZRANB3 recruitment to DNA damage sites upon UV irradiation. In contrast, expression of RHD-null (ΔRHD) TonEBP or Yc1 ΔRHD did not restore UV-induced ZRANB3 focus formation and ZRANB3 recruitment to DNA damage sites. Thus, TonEBP might be involved in template-switching DNA damage bypass pathway by promoting PCNA polyubiquitination and subsequent fork reversal.

### TonEBP Suppresses MMS-Induced Cell Death

**Figure 5:**
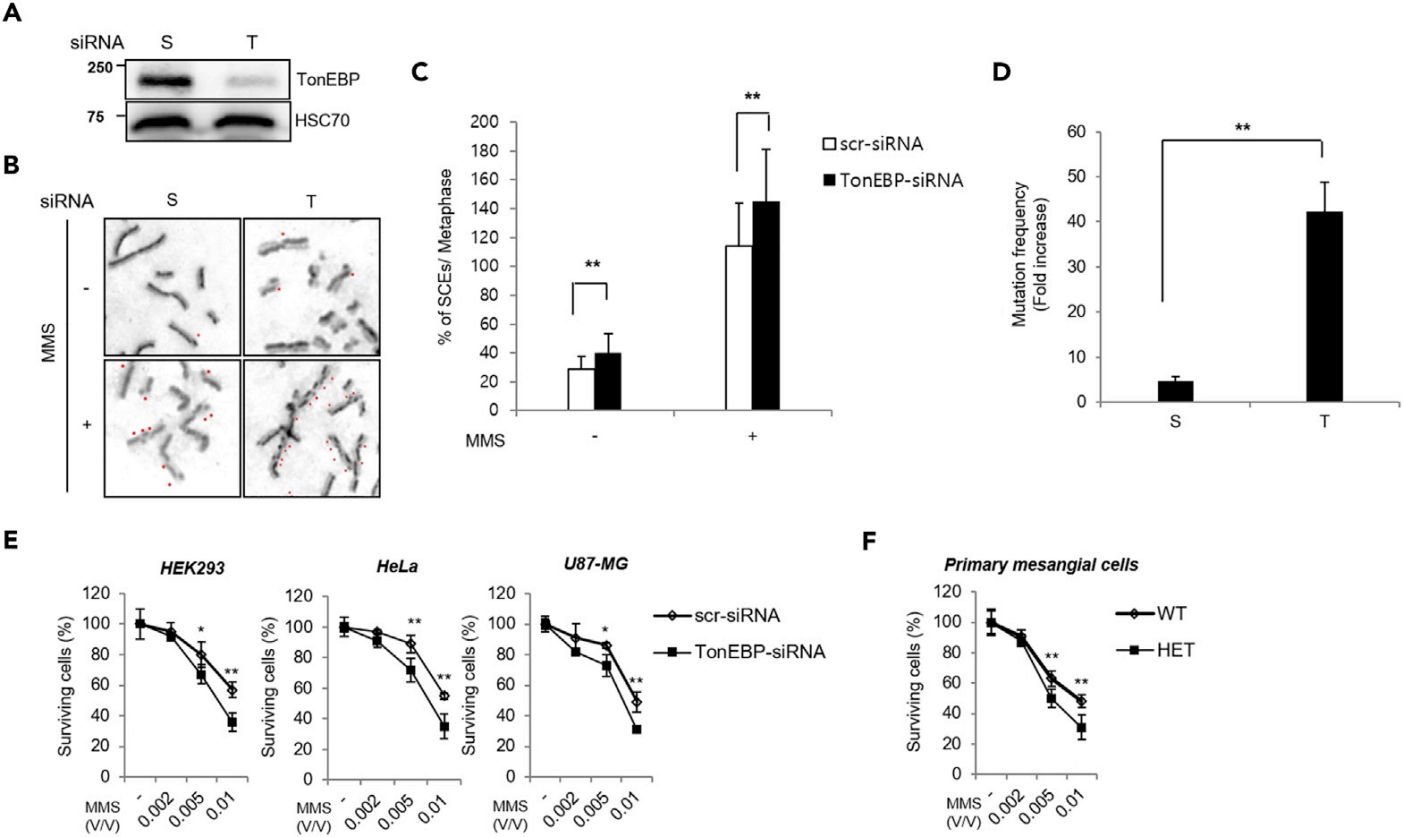
TonEBP Suppresses SCEs and Mutagenesis in Response to MMS Treatment (A) The TonEBP protein level was determined by western blot analysis. HEK293 cells were transfected with scrambled siRNA (S) or TonEBP-targeting siRNA (T). Whole-cell extracts were subjected for western blot analysis. (B) Representative images of SCEs. (C) For statistical analysis, SCEs after MMS treatment for 24 h were counted in 35 metaphase cells per group; mean ± SD, **p < 0.01. (D) HEK293 cells transfected with the indicated siRNAs were treated with 0.01% (v/v) MMS for 1 h, followed by incubation for 24 h. Mutation frequency was determined by the *SupF* plasmid mutagenesis assay; mean ± SD, **p < 0.01. (E) HEK293, HeLa, and U87-MG cell were transfected with the indicated siRNAs and treated with 0.01% (v/v) MMS. Live cells were counted after 24 h (mean ± SD, n = 4, ∗p < 0.05, ∗∗p < 0.01). (F) Primary cultures of renal mesangial cells were prepared from TonEBP haploinsufficient mice (HET) and their wild-type littermates (WT). Cells were treated with 0.01% (v/v) MMS, and live cells were counted (mean ± SD, n = 4. **p < 0.01).

The data presented above indicated that TonEBP participates in DNA damage tolerance by regulating PCNA polyubiquitination. Defects in DNA damage tolerance lead to increased sister chromatid exchange (SCEs), mutation frequency, and cell death in response to DNA damage. We therefore examined the effect of TonEBP deficiency on SCEs, mutagenesis, and cell survival in response to DNA damage. TonEBP knockdown significantly increased SCEs and mutation frequency in response to MMS (Figures 5A–5D). Accordingly, TonEBP knockdown had no effect on the survival of three cell lines (Figure 5E). Consistent with this finding, TonEBP haploinsufficiency did not affect cellular survival in primary cultures of renal mesangial cells (Figure 5F). TonEBP knockdown or TonEBP haploinsufficiency significantly decreased cell survival in response to MMS, and this effect was mediated by the induction of apoptosis (Figures S10A and S10B). MMS causes the formation of DNA adducts similar to those generated by UV irradiation (Marini and Wood, 2002, Minca and Kowalski, 2011, Reynolds et al., 2004). TonEBP knockdown or TonEBP haploinsufficiency reduced cell survival in response to UV irradiation (Figures S10C and S10D). By contrast, cell survival in response to HU or ionizing radiation (IR) was not affected by TonEBP knockdown (Figure S10E). Neither HU nor IR produces bulky DNA adducts (Koc et al., 2004, Rothkamm et al., 2003), suggesting that TonEBP mediates the tolerance to DNA damage associated with bulky DNA adduct formation. Collectively, these results meant that TonEBP prevents cell death, SCEs, and mutagenesis in response to DNA damage (in the form of bulky DNA adduct formation), consistent with its role in DNA damage tolerance.

### The RHD Is Essential for TonEBP Focus Formation and PCNA Polyubiquitination

**Figure 6:**
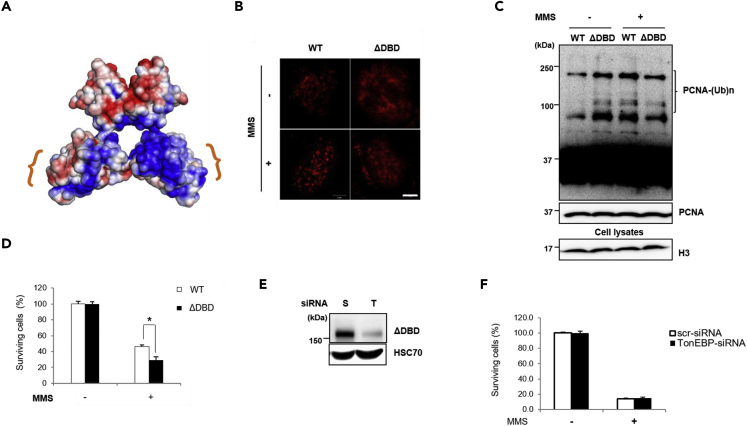
The RHD of TonEBP Is Required for MMS-Induced TonEBP Focus Formation, PCNA Polyubiquitination, and Cell Survival in Response to MMS Treatment (A) The deleted portion of the TonEBP RHD in the *TonEBP*^*Δ*^ allele is marked on the homodimer of TonEBP (PDB: 1IMH). (B) MEFs from *TonEBP*^*Δ/Δ*^ (ΔDBD) mice and their wild-type (WT) littermates were treated with 0.01% (v/v) MMS. TonEBP in the nucleus was visualized by immunostaining. Representative images of the nucleus are shown in each condition. Scale bar, 2 μm. (C) MEFs were treated with 0.01% (v/v) MMS, and chromatin-bound PCNA was detected as described in Figure 3A. (D) MEFs were treated with MMS at various doses as indicated, and live cells were counted; mean ± SD, n = 5, *p < 0.05. (E and F) ΔDBD MEFs were transfected with the indicated siRNAs. The cells were immunoblotted (E), and cell survival after MMS treatment was analyzed (F); mean ± SD, n = 3.

Last, we examined the importance of TonEBP RHD. We used the mouse TonEBP^Δ^ allele (ΔDBD), which represents the deletion of exons 6 and 7 of the *TonEBP* gene resulting in an in-frame deletion of the C-terminal portion of the RHD (Figure 6A) (Go et al., 2004). In contrast to the formation of TonEBP foci in wild-type mouse embryonic fibroblasts (MEFs) upon treatment with MMS, TonEBP foci were absent in ΔDBD MEFs (Figure 6B), suggesting that the RHD was required for TonEBP focus formation. RHD is also important for the interaction with SHPRH associated with PCNA polyubiquitination (Figure 2C). Accordingly, PCNA polyubiquitination after MMS treatment was significantly lower in ΔDBD MEFs (Figure 6C). ΔDBD MEFs manifested higher sensitivity to MMS treatment (Figure 6D), whereas the knockdown of TonEBP in ΔDBD MEFs (Figure 6E) did not further increase cell death after MMS treatment (Figure 6F). These findings indicated that the RHD of TonEBP is important for DNA damage tolerance.

**Figure 7:**
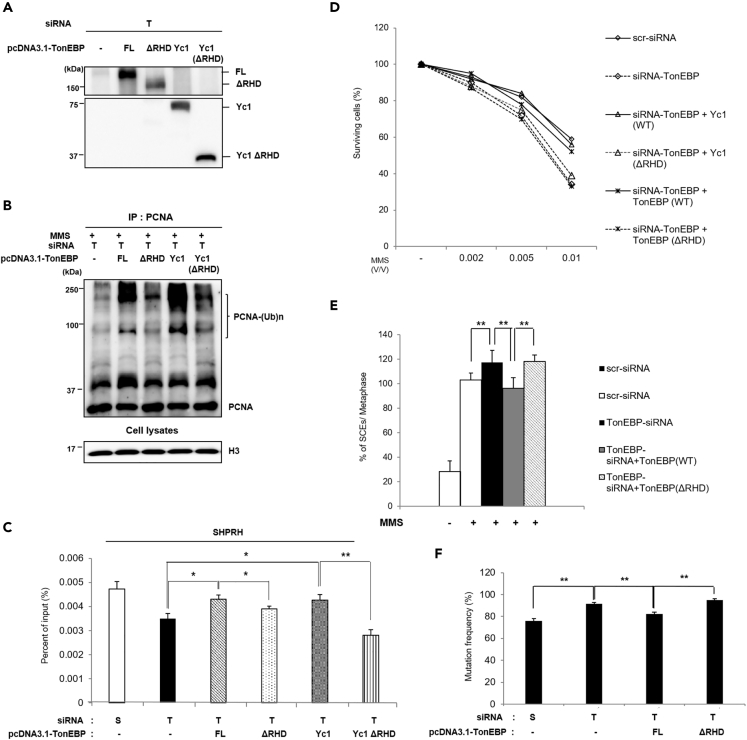
The RHD of TonEBP Is required for MMS-Induced PCNA Polyubiquitination, Cell Survival, and Genomic Stability in Response to MMS Treatment (A–F) HEK293 cells were transfected with TonEBP siRNA and a plasmid expressing full-length TonEBP (FL), TonEBP ΔRHD (ΔRHD), Yc1, or Yc1 ΔRHD. (A) Immunoblots of TonEBP. (B) PCNA polyubiquitination was analyzed in chromatin-bound fractions after MMS treatment. (C) SHPRH ChIP was performed as described in Figure 3D, mean ± SD, n = 3, ∗p < 0.05, ∗∗p < 0.01. (D) Cell survival after MMS treatment was measured; mean ± SD, n = 3. (E) SCEs were analyzed as described in Figure 5C; mean ± SD, **p < 0.01. (F) The SupF plasmid mutagenesis assay was performed as described in Figure 5D; mean ± SD, n = 3, **p < 0.01.

The role of RHD was confirmed by reconstitution experiments. PCNA polyubiquitination after MMS treatment was restored via ectopic expression of FL or Yc1 TonEBP (Figures 7A and 7B). Similarly, after MMS treatment, the recruitment of SHPRH to DNA damage sites in the *TP53* gene (Figure 7C) as well as cell survival (Figure 7D), SCEs (Figure 7E), and mutation frequency (Figure 7F) were restored by FL and Yc1 TonEBP. In contrast, ectopic expression of ΔRHD TonEBP or ΔRHD Yc1 TonEBP did not restore PCNA polyubiquitination or affect SHPRH recruitment to DNA damage sites, cell survival, SCEs, and mutation frequency after MMS treatment. Taken together, these results suggested that the RHD of TonEBP is necessary for the formation of TonEBP foci and for the regulation of PCNA polyubiquitination, whereas the C-terminal two-thirds of TonEBP are dispensable.

## Discussion

**Figure 8:**
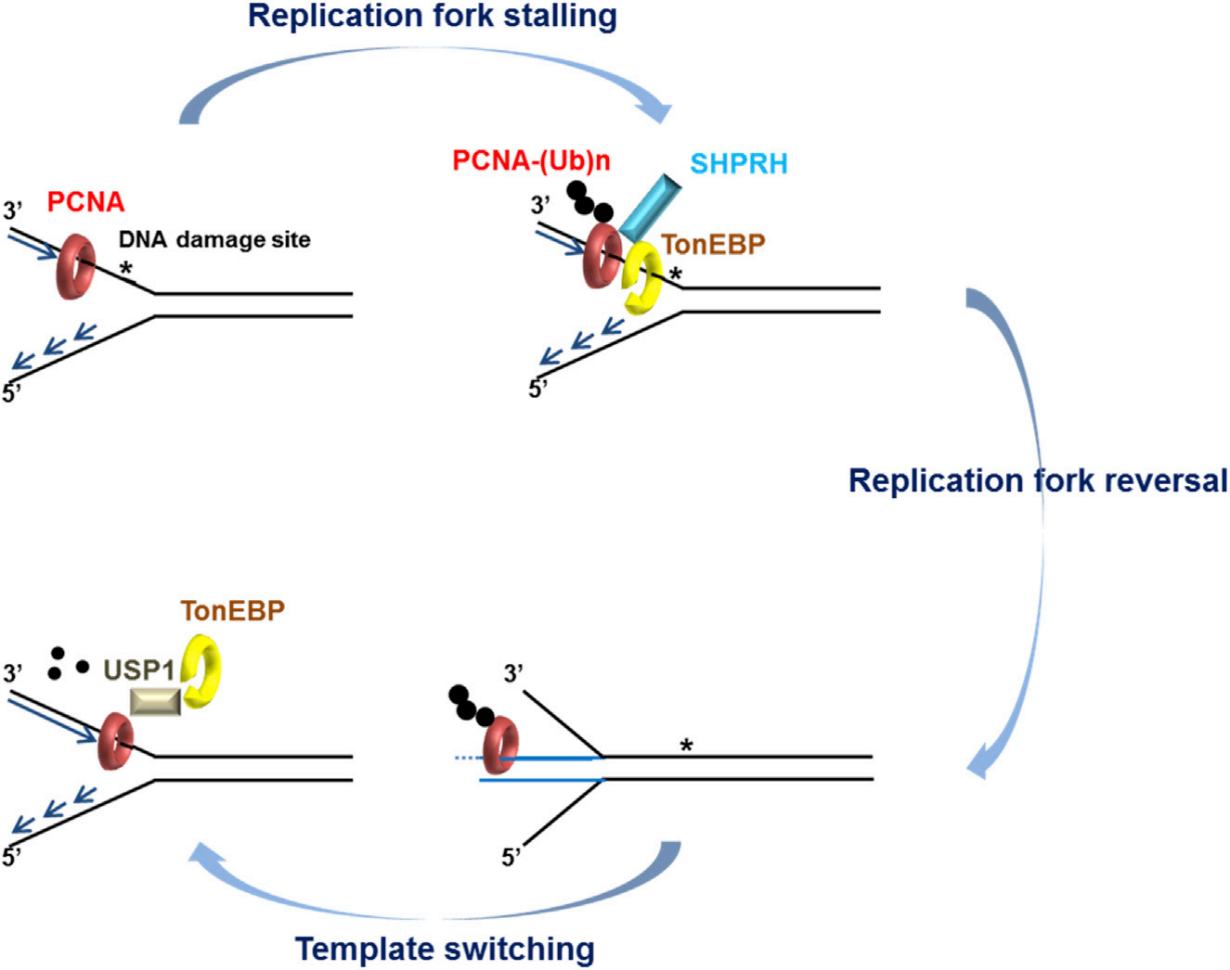
A Model of TonEBP Function in MMS-Induced PCNA Polyubiquitination Recruitment of TonEBP to DNA damage sites activates SHPRH to promote PCNA polyubiquitination through protein interactions. PCNA deubiquitination is necessary for the completion of fork reversal, and template switching is mediated by TonEBP-driven recruitment of USP1-UAF1 to DNA damage sites.

The RHD of TonEBP is distinct from other the RHDs of Rel proteins, which bind asymmetric DNA sites. TonEBP completely encircles its DNA target, and the DNA encirclement may increase the kinetic stability of the TonEBP-DNA complex (Stroud et al., 2002). We hypothesized that just as other proteins that encircle DNA (such as PCNA and minichromosome maintenance; Costa et al., 2011, De March and De Biasio, 2017), TonEBP participates in DNA surveillance. We found that TonEBP formed foci in response to MMS treatment and sequentially interacted with SHPRH and USP1, leading to PCNA polyubiquitination and subsequent deubiquitination. PCNA polyubiquitination triggers replication fork reversal in the DNA damage bypass pathway. Consistent with these data, TonEBP was required for replication fork protection presumably through fork reversal and DNA damage tolerance (Figure 8). The RHD of TonEBP was necessary for focus formation, regulation of PCNA polyubiquitination, and DNA damage tolerance, suggesting that DNA encirclement is essential for DNA damage surveillance by TonEBP.

PCNA polyubiquitination is necessary for DNA damage tolerance response because PCNA polyubiquitination induces replication fork reversal. Nonetheless, the mechanism by which DNA damage is detected leading to PCNA polyubiquitination by SHPRH and HLTF and deubiquitination by USP1 after the bypass has remained unclear. The present data suggest that TonEBP senses DNA damage sites with bulky adducts and sequentially recruits SHPRH to promote PCNA polyubiquitination and USP1 for PCNA deubiquitination. Therefore, TonEBP provides a platform for the recruitment of SHPRH and USP1 in a temporal manner to regulate PCNA polyubiquitination in response to DNA damage. The sequential recruitment of a ubiquitin ligase, SHPRH, and a deubiquitinating enzyme, USP1, to a DNA damage site by TonEBP might mediate the formation of a platform to initiate and finish fork reversal. ZRANB3, a polyubiquitin-recognizing protein, is recruited to the DNA damage sites for fork reversal (Ciccia et al., 2012). PCNA polyubiquitinated by TonEBP and SHPRH might allow ZRANB3 to move to DNA damage sites to protect stalled replication forks and induce fork reversal for template switching in the DNA damage bypass pathway (Vujanovic et al., 2017). After template switching for the DNA lesion bypass, the platform of polyubiquitinated PCNA is inactivated by TonEBP and USP1, displacing several proteins such as ZRANB3 from the DNA damage sites.

Defects in TonEBP caused sensitivity to MMS and UV irradiation, but not to HU or IR. UV irradiation and MMS produce bulky DNA adducts, which cause DNA replication stalling (Minca and Kowalski, 2011). Unlike the stimulatory effect of MMS on the levels of PCNA polyubiquitination, HU or IR treatment causes little or no PCNA polyubiquitination (Motegi et al., 2006, Motegi et al., 2008). Therefore, TonEBP might be a key sensor to promote PCNA ubiquitination (mono- to poly-) at bulky DNA adduct-related damage sites.

Proteins regulating gene transcription also function in other DNA processes such as DNA replication and repair. A recent study showed that RNA m^6^A (methylation of an A base), which is important for many RNA metabolic processes, is highly enriched at UV irradiation-induced DNA damage sites for Polκ recruitment (Xiang et al., 2017). SHPRH induces rRNA synthesis by recruiting RNA polymerase I to the rDNA promoter via the recognition of different histone methylation codes (Lee et al., 2017). In addition, DNA replication fork reversal can occur in response to high levels of transcription caused by a potential replication-transcription head-on collision (De Septenville et al., 2012, Trautinger et al., 2005). Therefore, proteins involved in transcription or replication repair may play a role in replication repair or transcription, respectively. Consistent with this idea, transcription factor TonEBP might take part in DNA damage bypass by regulating PCNA polyubiquitination to promote replication fork reversal.

There are a number of E3 ligases in the TonEBP interactome. In this study, we found that TonEBP selectively induces PCNA polyubiquitination through SHPRH after MMS-induced damage. We believe that UV light sensitivity caused by the TonEBP knockdown could be due to other E3 ligases interacting with TonEBP and presumably through PCNA polyubiquitination.

In summary, TonEBP regulates DNA damage tolerance through sequential interactions with SHPRH and USP1. The initial sensing of bulky DNA adducts by TonEBP and the provision of a platform for PCNA polyubiquitination upon MMS treatment potentially for fork reversal as well as the platform disassembly to end fork reversal constitute the initial and final molecular mechanisms of template switching. Because template switching mediated by fork reversal induced by PCNA polyubiquitination is an error-free model of DNA damage bypass, it could be a biomarker predicting the aggressiveness of tumors with different degrees of mutagenesis. This notion suggests that it is possible to select chemotherapeutic agents based on the expression level of TonEBP according to the mutagenesis burden of tumors.

### Limitations of the Study

In our study, only MMS was selected as a DNA-damaging agent inducing PCNA polyubiquitination. Although DNA-damaging agents other than MMS, e.g., UV light, HU, and IR, could induce DNA damage, our study revealed that TonEBP specifically performs its function in response to UV irradiation or MMS. Thus, further studies on UV light-induced PCNA polyubiquitination are needed. Nevertheless, because our study shows that UV-induced PCNA polyubiquitination rarely occurs in mammalian cells, we believe that TonEBP-dependent UV light-induced DNA damage response is minimal or has an alternative mechanism. Therefore, we are currently investigating a pathway independent of PCNA polyubiquitination in response to UV irradiation.

We proposed that TonEBP stabilizes a DNA replication fork in response to DNA damage. We suggested that this phenomenon may be mediated by fork reversal according to previous observations that PCNA polyubiquitination promotes fork reversal and some of our results. Nonetheless, we could not obtain direct evidence of fork reversal by electron microscopy owing to a technical issue. This topic should be investigated later.

## Methods

All methods can be found in the accompanying Transparent Methods supplemental file.

## Supplemental Information

Document S1. Transparent Methods, Figures S1–S10, and Tables S1 and S2
