# Supplementary material for: TonEBP Regulates PCNA Polyubiquitination in Response to DNA Damage through Interaction with SHPRH and USP1
Source: iScience. 2019 Jul 19;19:177–90. doi: 10.1016/j.isci.2019.07.021 (PMC6677787; doi:10.1016/j.isci.2019.07.021)
Supplement: Document S1. Transparent Methods, Figures S1–S10, and Tables S1 and S2 [file mmc1.pdf]

ISCI, Volume *19*

Supplemental Information

TonEBP Regulates PCNA Polyubiquitination in Response to DNA Damage through Interaction with SHPRH and USP1

Hyun Je Kang, Hyun Park, Eun Jin Yoo, Jun Ho Lee, Soo Youn Choi, Whaseon Lee-Kwon, Kyoo-young Lee, Jin-Hoe Hur, Jeong Kon Seo, Jae Sun Ra, Eun-A. Lee, Kyungjae Myung, and Hyug Moo Kwon

TonEBP regulates PCNA polyubiquitination in response to DNA damage through interaction with SHPRH and USP1

Supplementary Information

Hyun Je Kang[1], Hyun Park[1], Eun Jin Yoo[1], Jun Ho Lee[1], Soo Youn Choi[1], Whaseon Lee-Kwon[1], Kyoo-young Lee[2], Jin-Hoe Hur[3], Jeong Kon Seo[4], Jae Sun Ra[2], Eun-A Lee[2], Kyungjae Myung[1,2], and Hyug Moo Kwon[1]

[1]School of Life Sciences, Ulsan National Institute of Science and Technology, Ulsan 44919, Republic of Korea

[2]Center for Genomic Integrity, Institute for Basic Science, Ulsan 44919, Republic of Korea

[3]UNIST-Optical Biomed Imaging Center (UOBC), Ulsan National Institute of Science and Technology, Ulsan 44919, Republic of Korea

[4]UNIST Central Research Facilities (UCRF), Ulsan National Institute of Science and Technology, Ulsan 44919, Republic of Korea

Correspondence to Kyungjae Myung (lead contact): E-mail kmyung@ibs.re.kr; Phone +82 52-217-5323; Fax +82 52-217-5519 or Hyug Moo Kwon: E-mail hmkwon@unist.ac.kr; Phone +82 52-217-2535; Fax +82 52-217-3219

Transparent Methods

Cells and reagents

HEK293 and HeLa cells and MEFs were cultured in Dulbecco's Modified Eagle's Medium (DMEM) supplemented with 10% of fetal bovine serum (FBS; Thermo Fisher Scientific Inc., Waltham, MA, USA), penicillin, and streptomycin (100 U/ml and 100 µg/ml, respectively; GE Healthcare Life Sciences, Logan, UT, USA). Cells were maintained at 37°C in an incubator with 5% of CO_2. Cells were pretreated with MMS (Sigma-Aldrich, St. Louis, MO, USA) for various periods. Antibodies used for immunoblotting or IP were obtained from various companies (see Table S2). Cells were transfected with Lipofectamine 2000 or Lipofectamine RNAimax (Invitrogen, Carlsbad, CA, USA). siRNA duplexes were purchased from Integrated DNA Technologies (Coralville, IA, USA). Sources of all cells, reagents, and DNA constructs are listed in Table S2.

Immunofluorescence, microscopy, and image analysis

Cells were plated on LabTek chamber slides (Thermo Fisher Scientific) and incubated for 1 day before fixation with 100% methanol at 20°C for 30 minutes. For chromatin-bound proteins, cells were pretreated with 0.5% Triton X-100 for two minutes before fixation. For BrdU (Thermo Fisher Scientific) labeling, 20 µM BrdU was added for incubation for the variousperiods before fixation. For UV microirradiation, UVA laser (55 mW) irradiation was performed by means of a Palm MicroBeam laser microdissection workstation. The fixed cells were stained with the appropriate primary antibodies overnight at 4°C. After washes with 0.05% Triton X-100, Alexa Fluor–conjugated secondary antibodies were added and incubated for one hour. For BrdU detection, BrdU-labeled cells were fixed with 4% paraformaldehyde for five minutes after immunostaining. The cells were then treated with

4 N HCl for 10 minutes at room temperature (25℃). Next, the cells were washed with phosphate-buffered saline (PBS) and stained with a rat anti-BrdU antibody followed by Alexa Fluor–conjugated secondary antibody. The stained cells were mounted. Alexa Fluor 488–, 568–, and 633–conjugated secondary antibodies were purchased from Invitrogen.

TonEBP interactome analysis

To isolate TonEBP-interacting proteins, HEK293 cells were transfected with the pcDNA5/FRT empty vector or pcDNA5/FRT-TonEBP-Flag plasmid. The cells were lysed in 1 ml of RIPA buffer supplemented with a protease inhibitor cocktail and 0.5 mM phenylmethylsulfonyl fluoride at 24 hours post-transfection. Cell extracts were recovered by centrifugation at 12,000 rpm (Eppendorf, 5424R) for 15 minutes at 4°C. The cleared supernatants were collected, and the cell lysates were mixed with 50 ml (50% slurry) of anti-Flag M2 resin (Sigma-Aldrich) and incubated at 4°C for four hours. After centrifugation at 2500 rpm (Eppendorf, 5424R) for five minutes, the supernatants were discarded, and the collected resin samples were washed in 1 ml of RIPA lysis buffer five times. Flag-tagged TonEBP and bound proteins were eluted with excessive amounts of the 3× Flag peptide (Sigma-Aldrich). Eluted proteins were subjected to immunoblotting. The molecular identities of the eluted proteins were determined by mass spectrometry.

IP

For preparation of lysates for IP, cells were washed three times with ice-cold PBS and lysed in RIPA buffer as described previously (Lee et al., 2016). An appropriate antibody was added to lysates and incubated overnight at 4°C, followed by incubation with Protein A/G Sepharose beads (GE Healthcare Sciences). After extensive washing with RIPA lysis buffer, complexes were eluted and analyzed by immunoblotting.

Immunoblotting

Cell lysis for protein extraction was performed as described elsewhere (Lee et al., 2015). Protein concentration was measured with the BCA Protein Assay System (Pierce, Rockford, IL, USA). Equal amounts of protein from each sample were separated by SDS-PAGE and immunoblotted using specific primary antibodies. Horseradish peroxidase (HRP)-conjugated mouse, rabbit, and goat secondary antibodies were used for detection. The antigen–antibody binding was detected by means of enhanced chemiluminescence western blotting detection reagents (GE Healthcare Life Sciences).

Quantitative PCR

Quantitative PCR was performed using SYBR Green I Master and LightCycler 480 II (Roche, Rotkreuz, Switzerland). Measured threshold cycle (C_t) values were normalized to those of cyclophilin A.

ChIP

ChIP was performed as previously described (Lee et al., 2016). Briefly, HEK293 cells were treated with MMS for the various periods (Sigma-Aldrich), and chromatin was crosslinked with 1% formaldehyde before neutralization with 0.125 M glycine. After lysis and sonication (12 sonication cycles with Bioruptor NGS) to obtain DNA fragments between 250 and 500 bp), crosslinked chromatin was precleared with normal rabbit IgG or mouse IgG and Protein A Sepharose beads before IP with an anti-TonEBP, anti-SHPRH, or anti-USP1 antibody. Eluted protein–DNA complexes were washed extensively, and crosslinks in complexes were reversed by heating and proteinase K (Roche) treatment. Quantification of associated DNA was performed by quantitative PCR analysis with primers for *TP53* intron 7 (positions 13502–13724), forward primer: 5′-CCTCTTACCGATTTCTTCCA-3′ and reverse primer: 5′-AGGTCCTACCTGTCCCATTTA-3′, as described previously (Jiang

and Sancar, 2006).

Cell survival and apoptosis analysis

Cells grown in the presence or absence of MMS were trypsinized and counted at the various time points. For a terminal deoxynucleotidyl transferase dUTP nick-end labeling (TUNEL) assay, cells grown on coverslips were fixed in 4% formaldehyde and processed via the Apoptosis Detection System (Promega, Madison, WI, USA). Coverslips were analyzed under a LSM780NLO confocal microscope.

A SupF Plasmid Mutagenesis Assay

Control or TonEBP siRNA was introduced into HEK293T cells with RNAiMAX (Invitrogen). After 24 hours, shuttle plasmid pSP189 was cotransfected with control or TonEBP siRNA into HEK293T cells. After incubation at 37°C and 5% CO_2 for 24 hours, the cells were treated with 0.01% (v/v) MMS and allowed to recover in a fresh medium for another 24 hours. Plasmid DNA was isolated by means of the Spin Miniprep Kit (QIAGEN, Germantown, MD, USA). Recovered plasmid DNA was digested with Dpn I (Stratagene, La Jolla, CA, USA) and RNase I (Thermo Fisher Scientific), and introduced by heat shock into *Escherichia coli* MBM7070 carrying an amber mutation in the *lacZ* gene. After heat shock, the MBM7070 cell suspension was spread on Luria–Bertani (LB) agar plates with ampicillin, 5-bromo-4-chloro-3-indolyl-β-D-galactopyranoside (X-gal), and isopropyl-β-D-thiogalactoside. Mutation frequencies were measured by dividing the number of white colonies by the total number of colonies.

An SCE assay

HEK293T cells were cultured in the presence of 25 μg/ml BrdU for fourty eight hours. The cells were incubated for four hours with 0.2 μg/ml colcemid and then harvested by

trypsinization. The cells were then swollen in 75 mM KCl for 15 minutes at 37°C and fixed with methanol:acetic acid (3:1) twice. A drop of the cell suspension was placed onto glass microscope slides. The slides were treated with 1 μg/ml Hoechst 33258 (Invitrogen) for 30 minutes and then exposed to 265 nm UV light for 30 minutes. After that, the slides were incubated for 15 minutes in 2× SSC (Saline-sodium citrate buffer) at 65°C and then stained with a 5% Giemsa solution. Images were acquired using a fluorescence microscope (BX53; Olympus). At least 35 metaphase cells were chosen randomly from each group.

The molecular DNA combing assay

HEK293T cell were labeled for 30 minutes with 50 μM CldU (Sigma-Aldrich C6891) followed by 30 minutes of labeling with 250 μM IdU (Sigma-Aldrich I7125). To measure DNA replication rates, the cells were treated with 0.01% MMS and harvested. The cells were embedded in low-melting agarose (Bio-Rad, 161-3112) followed by DNA extraction. To stretch the DNA fibers, 22 × 22 mm silanized coverslips (Genomic Vision) were dipped into the DNA solution for 13 minutes and pulled out at a constant speed (300 μm/s) using Molecular Combing System (Genomic Vision MCS-001). The coverslips were baked for four hours at 60°C and incubated with 4N HCl for denaturation. CldU- and IdU-labeled tracts were detected by two hours incubation at room temperature (25℃) with a rat anti-BrdU antibody (dilution 1:100 detects BrdU and CldU; Abcam 6326) and a mouse anti-BrdU antibody (1:10, detects BrdU and IdU; Becton Dickinson 347580). The slides were fixed in 4% paraformaldehyde in PBS and incubated for one hour at room temperature (25℃) with an Alexa Fluor 488–conjugated goat anti-rat IgG antibody (dilution 1:100, A21208; Molecular Probes/Thermo Fisher) or an Alexa Fluor 568–conjugated goat

anti-mouse IgG antibody (dilution 1:100, A21124; Molecular Probes/Thermo Fisher). Finally, the coverslips were mounted with the ProLong Gold Antifade Reagent (Molecular Probes) and stored at −20°C. DNA fibers were examined under a Carl Zeiss microscope with Axio Observer 7 & ApoTome 2 (Motorized Fluorescence Microscope with Grid Projection) 63 objective. For each experiment, a total of 200 DNA fibers were analyzed, and the length of DNA fibers was measured in Adobe Photoshop.

Separation of nuclear and chromatin-bound fractions

The nuclear fraction was extracted using the Nuclear Extraction Kit (Pierce). The chromatin-bound fraction was extracted as described previously (Motegi et al., 2006).

Quantification and statistical analysis

All statistical analyses were performed by Student's *t* test. In figures, n means the number of biological replicates.

References

Jiang, G., and Sancar, A. (2006). Recruitment of DNA damage checkpoint proteins to damage in transcribed and nontranscribed sequences. Molecular and cellular biology *26*, 39-49.

Lee, H.H., Sanada, S., An, S.M., Ye, B.J., Lee, J.H., Seo, Y.K., Lee, C., Lee-Kwon, W., Kuper, C., Neuhofer, W.*, et al.* (2016). LPS-induced NFkappaB enhanceosome requires TonEBP/NFAT5 without DNA binding. Scientific reports *6*, 24921.

Lee, J.H., Lee, H.H., Ye, B.J., Lee-Kwon, W., Choi, S.Y., and Kwon, H.M. (2015). TonEBP suppresses adipogenesis and insulin sensitivity by blocking epigenetic transition of PPARgamma2. Scientific reports *5*, 10937.

Motegi, A., Sood, R., Moinova, H., Markowitz, S.D., Liu, P.P., and Myung, K. (2006). Human SHPRH suppresses genomic instability through proliferating cell nuclear antigen polyubiquitination. The Journal of cell biology *175*, 703-708.

Supplementary figure legends

Figure S1. TonEBP interacts with SHPRH, USP1, HLTF, and UAF1, related to Figure 1

(A) HeLa cells treated with 60 J/m^2 ultraviolet irradiation (UV), 2.5 mM hydroxyurea (HU), or 60 ng/ml mitomycin C (MMC) for 30 minutes were fixed and immunostained with the anti-TonEBP antibody. Representative images of the nucleus are shown for each group. Scale bar, 2 μm. (B) HEK293 cells were transfected with plasmids expressing proteins Flag-HA-TonEBP and Myc-SHPRH. Cell lysates were subjected to IP with the anti-HA antibody. Precipitated proteins were detected with the various antibodies. (C) HEK293 cells were transfected with plasmids expressing Flag-HA-TonEBP and Myc-USP1. Cell lysates were subjected to IP with the anti-HA antibody. Precipitated proteins were detected with the various antibodies. (D) HEK293 cells were transfected with plasmids expressing Myc-SHPRH and Flag-USP1. Cell lysates were subjected to IP with the anti-Flag antibody. Precipitated proteins were detected with the various antibodies. (E) HEK293 cells were transfected with plasmids expressing Flag-HA-TonEBP and Myc-UAF1. Cell lysates were subjected to IP with the anti-HA antibody. Precipitated proteins were detected with the various antibodies. (F) HEK293 cells were transfected with plasmids expressing proteins Flag-TonEBP, Myc-SHPRH, and HA-USP1. Cell lysates were subjected to IP with the anti-Flag antibody. Precipitated proteins were analyzed with the various antibodies. Benzonase (2500 U) was added during the IP reaction to eliminate indirect interactions through DNA.

Figure S2. Heterodimerization of Yc1 with endogenous FL TonEBP does not affect the interaction with SHPRH and USP1, related to Figure 2

(A) HEK293 cells were transfected with TonEBP-targeting siRNA (T) followed by plasmids expressing Myc-tagged SHPRH together with Flag-tagged Yc1, Yc1 ΔIPT, or Yc1 ΔRHD. The proteins immunoprecipitated with the anti-Myc antibody were detected with the anti-Flag antibody. (B) HEK293 cells were transfected with TonEBP-targeting siRNA (T)

followed by a plasmid expressing HA-USP1 together with a plasmid expressing Flag-tagged Yc1, Yc1 ΔIPT, or Yc1 ΔRHD. The proteins immunoprecipitated with the anti-HA antibody were detected with the anti-Flag antibody.

Figure S3. Effects of SHPRH or USP1 on PCNA polyubiquitination, related to Figure 3

(A) HEK293 cells transfected with scrambled (control) siRNA or SHPRH-targeting siRNA were treated with 0.01% (v/v) MMS for one hour as indicated. PCNA bound to chromatin was immunoprecipitated and detected with the anti-PCNA antibody after four hours. (B) HEK293 cells transfected with scrambled siRNA or USP1-targeting siRNA were treated with 0.01% (v/v) MMS for one hour as indicated. PCNA bound to chromatin was immunoprecipitated and detected with the anti-PCNA antibody after four hours.

Figure S4. Effects of SHPRH or USP1 effects on TonEBP amount–dependent PCNA polyubiquitination, related to Figure 3

(A) HEK293 cells transfected with TonEBP-targeting siRNA (T) and SHPRH-targeting siRNA (S) followed by TonEBP-expressing plasmids at various doses as indicated were treated with 0.01% (v/v) MMS for one hour. PCNA bound to chromatin was immunoprecipitated and detected with the anti-PCNA antibody. (B) HEK293 cells transfected with TonEBP-targeting siRNA (T) and USP1-targeting siRNA (U) followed by TonEBP-expressing plasmids at various doses as indicated were treated with 0.01% (v/v) MMS for one hour. PCNA bound to chromatin was immunoprecipitated and detected with the anti-PCNA antibody.

Figure S5. mRNA expression levels of *SHPRH* and *USP1* are not affected by TonEBP, related to Figure 3

(A) HEK293 cells were transfected with the indicated siRNAs, and proteins were detected with the indicated antibodies. (B) HEK293 cells were transfected with the indicated siRNAs and treated with 0.005% (v/v) MMS for one hour, followed by incubation for 24 hours. Proteins were detected with the indicated antibodies. S and T indicate scrambled siRNA and TonEBP siRNA.

Figure S6. Effects of TonEBP on UV light–induced PCNA polyubiquitination, related to Figure 3

(A) HEK293 cells transfected with scrambled siRNA (S) or TonEBP-targeting siRNA (T) were irradiated with 60 J/m^2 UV light. PCNA bound to chromatin was immunoprecipitated and detected with the anti-PCNA antibody after four hours. (B) HEK293 cell lysates were subjected to IP with the indicated antibodies. Cells treated with normal serum (Serum) served as a negative control. The anti-TonEBP antibody was employed for IP. Precipitated proteins were detected with the indicated antibodies. (C) HEK293 cells were transfected with scrambled siRNA or SHPRH-targeting siRNA. Cell lysates were subjected to IP with the indicated antibodies. Cells treated with normal serum (Serum) served as a negative control. The anti-TonEBP antibody was used for IP. Precipitated proteins were detected with the indicated antibodies.

Figure S7. The TonEBP–SHPRH–USP1 complex, related to Figure 3

HEK293 cells were transfected with the plasmids expressing proteins HA-TonEBP, Myc-SHPRH, and Flag-USP1. Cell lysates were subjected to IP with the anti-Flag or anti-Myc antibody. Precipitated proteins were detected with the indicated antibodies.

Figure S8. TonEBP, SHPRH, and USP1 form a complex, and TonEBP is recruited to DNA damage sites, related to Figure 3

(A) Location of DNA damage sites in intron 7 of the *TP53* gene. The region from nucleotide position 13502 to 13724 was amplified by quantitative PCR for ChIP. (B, C) HEK293 cells were incubated without MMS (-), or with 0.01% (v/v) MMS for one hour, followed by incubation for a half or one hour as indicated. ChIP was performed using anti-ATR and anti-RPA32 antibodies (B) and the anti-TonEBP antibody as indicated (C). The percentage of input in the precipitates is shown. Mean ± SD, n=3

Figure S9. Specificity of the anti-ZRANB3 antibody and the necessity of TonEBP for MMS-induced replication fork protection, related to Figure 4

(A) U2OS cells were transfected with scrambled siRNA or ZRANB3-targeting siRNA. After that, proteins were detected with the indicated antibodies. (B) HeLa cells were transfected with scrambled siRNA or TonEBP-targeting siRNA. The cells were then pulse-labeled with 20 µM BrdU for 10 minutes and treated with 0.01% (v/v) MMS for one hour before fixation and immunostaining for BrdU. Representative images of the nucleus inside white lines are presented for each group. Scale bar, 2 µm. (C) BrdU signal intensity in each nucleus was measured in 200 cells from each group. Mean + SD, *p < 0.05, **p < 0.01.

Figure S10. TonEBP promotes cell survival after treatment with UV light but not HU or IR, related to Figure 5

(A) HeLa cells were transfected with the indicated siRNAs and treated with 0.01% (v/v) MMS. The TUNEL assay was performed, and cells were stained with propidium iodide (PI). Arrowheads indicate cells positive for PI and TUNEL signals. Representative images of the nucleus inside white lines are shown for each group. Scale bar, 20 µm. (B) Percentages of double-positive cells were measured among 300 cells from each group.

Mean ± SD, $^*p < 0.05$. (C) HeLa cells were transfected with the indicated siRNAs and treated with 60 J/m^2 UV irradiation. Live cells were counted after 24 hours. Mean ± SD, n = 5, $^*p < 0.05$. (D) Primary cultures of renal mesangial cells were derived from TonEBP haploinsufficient mice (HET) and their wild-type littermates (WT). Mean ± SD, n = 5, $^*p < 0.05$, $^{**}p < 0.01$. Cells were treated with 60 J/m^2 UV irradiation, and live cells were counted after 24 hours. (E) HeLa cells were transfected with the indicated siRNAs and treated for 24 hours with 2.5 mM HU or 10 Gy IR. Live cells were counted. Mean ± SD, n = 5
